# Professor Umberto Veronesi: a physician, a researcher, a brilliant man

**DOI:** 10.3332/ecancer.2017.742

**Published:** 2017-06-08

**Authors:** Ketti Mazzocco, Chiara Marzorati, Gabriella Pravettoni

**Affiliations:** 1 Applied Research Division for Cognitive and Psychological Science, European Institute of Oncology (IEO), Via Ripamonti 435, Milan 20139, Italy; 2 Department of Oncology and Haemato-Oncology (DIPO), University of Milan, Via Festa del Perdono, Milan, Italy; 3 Foundations of the Life Sciences, Bioethics and Cognitive Sciences, European School of Molecular Medicine (SEMM), European Institute of Oncology, Via Adamello 16, Milan 20139, Italy

**Keywords:** breast cancer, patient-centred care, psychosocial aspects, medical decision-making, Umberto Veronesi

## Abstract

Many of those who work in oncology or deal with cancer patients know of Prof. Umberto Veronesi and none of them could deny the importance of his battle against cancer.

He devoted his life to improving cancer treatment and quality of life for patients. He was a physician, and a politician, but above all he was a researcher. He embodied the true spirit of research, i.e., to believe in something and investigate every aspect of it until all the questions about it have been satisfactorily answered. He never gave up when faced with challenges, and he never stopped being curious. He believed in science, because he wanted to believe in the future. He mixed scientific knowledge with human warmth and was the pioneer of many breast cancer innovations.

From the beginning of Prof. Veronesi’s career, his mission was clear: ‘*My first decision was to focus on the fight against cancer. When I started at the National Cancer Institute in Milan, I felt a profound sense of rebellion against the surrender of doctors and patients to a disease that caused intense suffering. In particular, I could not stand the havoc of a woman’s body after a mastectomy: in order to remove just a small breast cancer, not only the breast was taken away from the body, but also the axillary lymph nodes and the chest muscles. Consequently, I decided to fight mainly against breast cancer. It was a tough war: the dogma of mastectomy was so deeply-rooted that everybody thought I was crazy when I suggested conservative breast surgery.*’

Prof Veronesi therefore carried out a randomised research study where 800 women with small breast cancers (less than 2 cm diameter) were enrolled: half were treated with Halsted radical mastectomy and half with quadrantectomy, a new breast conserving surgery which removed ‘a quarter of the breast.’ After eight years, he demonstrated that there was no significant difference in recurrence between the two groups [1–2]. The equal therapeutic result of quadrantectomy axillary dissection and mastectomy, with a better quality of life (QoL) in the case of conservative surgery, was confirmed also by other researchers shortly after Veronesi’s findings [3]. A 20-year follow-up study further supported the efficacy and safety of this innovative therapeutic option: the overall and breast cancer-specific survival rates were similar in the two groups [4].

The quadrantectomy was just one of the visionary treatments implemented by Veronesi in order to save women’s lives and, moreover, to improve their QoL. Sentinel node biopsy permitted avoidance of axillary dissection if the sentinel node was disease-free, while intraoperative radiotherapy and nipple-sparing techniques were other treatments aimed at obtaining better aesthetics instead of scarred results.

These innovations contributed to a paradigm shift: ensuring the maximum efficacy with minimal invasiveness and consequent side effects. In this perspective, we assisted to a shift from maximum tolerated dose to minimum effective dose, from the strongest treatment that patients could tolerate to the maximum efficacy with minimal drug, surgery, or irradiation induced side effects. The minimum effective dose could be considered a benchmark of Veronesi’s struggle that contributed to the response of his main concerns: the centralisation of the patient and the way towards personalised medicine. Taking into account psychological and social aspects, as well as the physical condition, was what Umberto Veronesi wanted to teach to all his students and colleagues.

Today, breast cancer is the most frequent cancer in women with 1.67 million new cases worldwide, and during the last 15 years, cancer has been the third cause of death in the world [5, 6]. These are not mere numbers. Behind them, there are people with children and families, there are caregivers or offspring, and there are women or men with a life that they want to live. Veronesi’s aim was to beat cancer, but also to alleviate patients’ suffering.

Recent articles demonstrated how physical and psychological symptoms have to be managed during the process of care in order to prevent the onset of unmet needs after treatment [7]. Among which the most common are fatigue, sexuality, weight and concentration, besides the lack of information about the patient’s own clinical condition and about what can happen during and after treatment. Worse psychosocial adaptation, including QoL, patient concerns, anxiety, and depression, correlates with higher unmet needs. In addition, treatments for the illness are physically demanding (often life threatening) both for disability and pain or fatigue, and they obviously contribute to the occurrence of emotional and mental health problems [8]. Women with breast cancer go through different treatments, some of which can cause detrimental effects to self-confidence and intimate relationships, contributing to the emergence of a dissonance between women and their body image accompanied by a loss of the sense of femininity, motherhood, sexuality, and increased psychological distress [9, 10].

These psychosocial aspects cannot be cured only with drug discoveries or surgical interventions. We need to change our point of view and follow what Umberto began: ‘*To cure the disease in the body is not enough. Healing the mind is equally necessary. If the disease is hidden somewhere in our head, there it will remain for a lifetime. Nowadays, it is easy to remove a lump from the breast of a woman, but it is more difficult to get it out of her mind. Even though many years have passed, the memory of cancer can haunt, make fears and, anxieties arise and therefore create unhappiness. As Plato said, the body and soul go together. Treating only the first one is not enough.*’

It is fundamental to put people at the heart of our work, because they are the heart of our healthcare activity.

*In the last century, we have committed to evidence-based medicine, but in the new millennium, we need to restore humanity*. We need to improve a model that has already been implemented in cancer care and which Veronesi made his guiding principle: patient-centred care—a model that decreases the psychological impact for equal oncological efficacy.

Patient-centred care is characterised by the recognition of patient needs and the incorporation of each individual patient’s perspective in medical decision-making. A good patient-physician relationship is essential to create better understanding of what patients need, and it is an investment of confidence, a need to share, without hesitation and defences, a common ground that will allow us to communicate as equals. Empathy, trust, and information are the pillars for helping people with cancer to manage their disease. The need to know and the way in which physicians communicate are important determinants to make an informed shared decision about treatments in breast and all cancers in women [11, 12]. It is also important that women do not feel different after the disease because they feel betrayed by their own body: everything that we do not process returns. If we do not learn to understand how we can live well or give the right weight to what we feel, we run the risk of remaining imprisoned in a state of suffering and fear, paralysed and helpless.

The disease could be more or less severe on the basis of how our mind perceives it. For this reason, how we communicate plays an important role during every moment of the care process. We need to know who our patients are, and what they think or hope. We need to remind them that they are not their disease, and we have to help them turn dramatic moments into opportunities of rebirth.

We should overcome the word ‘patient,’ which may indicate a passive and resigned attitude and start speaking about ‘people with.’ Recent studies emphasise people’s desire to actively participate in their treatment choices: people with breast, prostate, colorectal, lung, gynaecological, and other cancers prefer a more shared or active role versus a more passive role [13].

We must carry out Umberto’s legacy and pursue the humanisation of care. We should draw strength from his foresight to implement more humane, efficient, and accessible treatments. We must get to know our patients as people not as bodies to heal. Beating cancer is not enough, and we need to process all the aspects of that experience to make it useful. The disease is a powerful experience that marks profoundly those who go through it, and it could help them to be stronger at the most difficult times. Cancer survivors have scars that are invisible to others, but this is what makes them more aware about their lives. According to a Japanese tradition, if you break a vase, you put the pieces together with gold in order to make it more valuable. We can use this metaphor to for cancer in a person’s life: it is the break in the vase, it is like a scar, and it can destroy the body and the soul, but if we are able to heal the wound, a greater beauty will be reborn. We should go beyond suffering in order to be able to enjoy a new way of life. The signs of life on our skin and in our mind have value and meaning, and from their acceptance and healing, regeneration processes and inner rebirth can kick off, making us new and resolute people. We should always remember, as Umberto said, that ‘*people with cancer are not only patients, but people who have to be treated in the body and soul as curing the husk is not enough.*’
